# The “Angiogenic Switch” and Functional Resources in Cyclic Sports Athletes

**DOI:** 10.3390/ijms22126496

**Published:** 2021-06-17

**Authors:** Olga V. Balberova, Evgeny V. Bykov, Natalia A. Shnayder, Marina M. Petrova, Oksana A. Gavrilyuk, Daria S. Kaskaeva, Irina A. Soloveva, Kirill V. Petrov, Elena Y. Mozheyko, German V. Medvedev, Regina F. Nasyrova

**Affiliations:** 1 Research Institute of Olympic Sports, Ural State University of Physical Culture, 454091 Chelyabinsk, Russia; bev58@yandex.ru; 2 V.M. Bekhterev National Medical Research Center for Neurology and Psychiatry, Department of Personalized Psychiatry and Neurology, 192019 Saint Petersburg, Russia; 3 Department of Outpatient Therapy and Family Medicine with a Postgraduate Course, Shared Core Facilities Molecular and Cell Technologies, Professor V.F. Voino-Yasenetsky Krasnoyarsk State Medical University, 660022 Krasnoyarsk, Russia; stk99@yandex.ru (M.M.P.); dashakas.ru@mail.ru (D.S.K.); 4 The Department of Polyclinic Therapy and Family Medicine and Healthy Lifesttyle with a Course of PE, V. F. Voino-Yasenetsky Krasnoyarsk State Medical University, 660022 Krasnoyarsk, Russia; oksana.gavrilyuk@mail.ru; 5 Department of Hospital Therapy and Immunology with a Postgraduate Course, Shared Core Facilities Molecular and Cell Technologies, Professor V.F. Voino-Yasenetsky Krasnoyarsk State Medical University, 660022 Krasnoyarsk, Russia; solovieva.irina@inbox.ru; 6 Department of Physical and Rehabilitation Medicine with a Postgraduate Course, Shared Core Facilities Molecular and Cell Technologies, Professor V.F. Voino-Yasenetsky Krasnoyarsk State Medical University, 660022 Krasnoyarsk, Russia; kllpetrov@mail.ru (K.V.P.); el_mozhejko@mail.ru (E.Y.M.); 7 R. R. Vreden National Medical Research Center for Traumatology and Orthopedics, Department of Hand Surgery with Microsurgical Equipment, 195427 Saint-Petersburg, Russia; dr.medvedev.g@yandex.ru

**Keywords:** angiogenic switch, angiogenesis, vascular endothelial growth factor (VEGF), fibroblast growth factor (FGF), angiopoietin, platelet growth factor (EDGF), epidermal growth factor (EGF), receptor, skeletal muscle, myocardium, lung tissue, nervous tissue, pathology, physical activity, sports

## Abstract

Regular physical activity in cyclic sports can influence the so-called “angiogenic switch”, which is considered as an imbalance between proangiogenic and anti-angiogenic molecules. Disruption of the synthesis of angiogenic molecules can be caused by local changes in tissues under the influence of excessive physical exertion and its consequences, such as chronic oxidative stress and associated hypoxia, metabolic acidosis, sports injuries, etc. A review of publications on signaling pathways that activate and inhibit angiogenesis in skeletal muscles, myocardium, lung, and nervous tissue under the influence of intense physical activity in cyclic sports. **Materials**: We searched PubMed, SCOPUS, Web of Science, Google Scholar, Clinical keys, and e-LIBRARY databases for full-text articles published from 2000 to 2020, using keywords and their combinations. **Results**: An important aspect of adaptation to training loads in cyclic sports is an increase in the number of capillaries in muscle fibers, which improves the metabolism of skeletal muscles and myocardium, as well as nervous and lung tissue. Recent studies have shown that myocardial endothelial cells not only respond to hemodynamic forces and paracrine signals from neighboring cells, but also take an active part in heart remodeling processes, stimulating the growth and contractility of cardiomyocytes or the production of extracellular matrix proteins in myofibroblasts. As myocardial vascularization plays a central role in the transition from adaptive heart hypertrophy to heart failure, further study of the signaling mechanisms involved in the regulation of angiogenesis in the myocardium is important in sports practice. The study of the “angiogenic switch” problem in the cerebrovascular and cardiovascular systems allows us to claim that the formation of new vessels is mediated by a complex interaction of all growth factors. Although the lungs are one of the limiting systems of the body in cyclic sports, their response to high-intensity loads and other environmental stresses is often overlooked. Airway epithelial cells are the predominant source of several growth factors throughout lung organogenesis and appear to be critical for normal alveolarization, rapid alveolar proliferation, and normal vascular development. There are many controversial questions about the role of growth factors in the physiology and pathology of the lungs. The presented review has demonstrated that when doing sports, it is necessary to give a careful consideration to the possible positive and negative effects of growth factors on muscles, myocardium, lung tissue, and brain. Primarily, the “angiogenic switch” is important in aerobic sports (long distance running). Conclusions: Angiogenesis is a physiological process of the formation of new blood capillaries, which play an important role in the functioning of skeletal muscles, myocardium, lung, and nervous tissue in athletes. Violation of the “angiogenic switch” as a balance between proangiogenic and anti-angiogenic molecules can lead to a decrease in the functional resources of the nervous, musculoskeletal, cardiovascular, and respiratory systems in athletes and, as a consequence, to a decrease in sports performance.

## 1. Introduction

In the modern world, sports are considered as one of the components of a healthy lifestyle, including prevention of cerebral ischemia, coronary heart disease, diabetes mellitus, atherosclerosis, and many other diseases. However, angiogenesis, as a dynamic and complex process regulated by various growth factors, can change under the influence of active physical activity. Changes can occur both in the positive direction and in the direction of the development of the pathological process. Against this background, it is promising and important to study the so-called “angiogenic switch” as an imbalance between proangiogenic and anti-angiogenic molecules (inhibitors and activators of angiogenesis) [1]. The “angiogenic switch”, through which new capillaries grow in the vasculature, includes the prevascular phase and the vascular phase. In the prevascular phase, germinating cells proliferate (sometimes as quickly as in the vascular phase), but the rate of these cells’ death (apoptosis) balances proliferation and maintains the germinating mass in a stable state. In the vascular phase, exponential cell growth begins [2]. The change in the activity of the synthesis of angiogenic molecules depends on an increase in the production of one or several angiogenesis activators such as endothelial vascular growth factor (VEGF), fibroblast growth factor (FGF), platelet growth factor (PDGF), epidermal growth factor (EGF), and others. They can be mobilized from the extracellular matrix or released from host cells (for example, macrophages). The expression of endogenous angiogenesis inhibitors such as thrombospondin-1 (TSP-1) or interferon beta can be suppressed. Thus, the “angiogenic switch” includes something more than a simple activation of angiogenic activity and is considered to be the result of a balance of positive and negative regulators [2]. Integrin signaling also contributes to this regulatory balance. Resting vessels express one class of integrins, while germinating capillaries express another. Interference with signaling from the latter class of integrins can inhibit angiogenesis. Proteases control the bioavailability of angiogenic activators and inhibitors. Some release FGF-2 stored in the extracellular matrix, while plasmin, a proangiogenic component of the blood coagulation system, is degraded to form an angiogenesis inhibitor, angiostatin [3]. Activation of the angiogenic process can be caused by local changes in tissues under the influence of excessive physical exertion and their consequences, such as hypoxia, metabolic acidosis, trauma, inflammation, and impaired homeostasis of metal ions. The study of angiogenesis and the “angiogenic switch” in athletes and the translation of the results of these studies into the training process is important for maintaining the physical health of athletes as well as increasing the functional resources of the nervous, musculoskeletal, cardiovascular, and respiratory systems of athletes and their sports performance.

## 2. Materials

We searched for articles for the period from 2000 to 2020 in the e-LIBRARY, SCOPUS, Web of Science, Google Scholar, Clinical keys, and PubMed databases using the keywords: angiogenic switch, angiogenesis, vascular endothelial growth factor (VEGF), growth factor fibroblasts (FGF), angiopoietin, platelet growth factor (EDGF), epidermal growth factor (EGF), receptor, skeletal muscle, myocardium, lung tissue, nervous tissue, pathology, physical activity, sports.

## 3. Results

### 3.1. Vascular Endothelial Growth Factor (VEGF)

#### 3.1.1. Role of VEGF in Angiogenesis in Athletes

An important aspect of adaptation to training loads in cyclic sports is an increase in the number of capillaries in muscle fibers, which improves the metabolism of skeletal muscles and myocardium, as well as in the brain and lung tissue. Primarily, the “angiogenic switch” is important in aerobic sports (i.e., long distance running). The high capillarization of the working muscles increases the peripheral circulation and thus influences the maximum oxygen consumption. This is the key to the high athletic performance of long distance runners (endurance training). Vasculogenesis (the first stage is the primitive formation of vessels inside the embryo and in the surrounding membranes, as well as the circulation of the yolk sac) and angiogenesis (second stage is responsible for the remodeling and expansion of this network) the formation of new blood vessels) play an important homeostatic role, as blood vessels carry nutrients to tissues and organs and remove catabolic products. However, uncontrolled growth of blood vessels can contribute to the development of many pathological processes, including vascular disorders [4]. From this point of view, an important aspect of adaptation to sports loads is the establishment of a balance in the synthesis of proangiogenic and anti-angiogenic molecules (activators and inhibitors of angiogenesis), or the “angiogenic switch”. Identification of genetic markers associated with the regulation of blood vessel growth is an important aspect in sports practice. One of the key factors affecting vascular growth is the VEGF-A protein. It is a heparin-binding protein that exists as a disulfide-linked homodimer. This growth factor causes proliferation and migration of vascular endothelial cells and is involved in the regulation of both physiological and pathological angiogenesis. VEGF-A expression is significantly increased in endurance athletes (for example, in long distance runners) [5].

##### VEGF and the “Angiogenic Switch” in Skeletal Muscle

Muscle injuries are one of the most common pathologies in orthopedic practice. Most muscle injuries (10% to 55% of all injuries) occur during sports activities. Muscle injuries are a serious problem due to slow recovery, during which athletes are unable to participate in training and competition, as well as due to the frequent consequences and recurrence of injuries [6]. Muscle regeneration is a complex biological process. Once damaged, satellite cells proliferate, thus providing enough cells for repair. The proliferation phase ends with the appearance of the first small regenerating muscle tubes approximately 3 days after injury [7]. At the same time, some of the proliferated cells become immobile again, while the rest begin to merge with the formation of multinucleated muscular tubes (terminal differentiation). Subsequently, there is a biochemical differentiation of muscle tubes into muscle fibers. This process is accompanied by an increase in macrophages and the destruction of damaged fibers. The results obtained when studying the effect of VEGF-A on the formation of myotubes show that this process is associated with the stimulation of differentiation by increasing the maturation of multinucleated myofibrils [8]. To identify the receptor that mediates these effects of VEGF-A on myogenic cells, it must be kept in mind that both VEGFR-1 and VEGFR-2 are activated in differentiating myogenic cells, in myotubes, and in satellite cells after muscle injury. In cultured myoblasts and cells, inhibition of the VEGFR-2 protein kinase reverses the protective effect of VEGF-A on cell apoptosis, suggesting the involvement of VEGFR-2 in mediating VEGF-A signal. The effect of VEGF-A on muscle recovery after injury is likely to be mediated by various mechanisms. The factor has a well-known proangiogenic activity; research results show [9,10] that it prevents apoptosis and promotes muscle fiber growth. According to the authors, the restoration of muscle tissue is realized through the interaction of all the listed effects of VEGF-A. An additional possibility is that mobilizing bone marrow progenitor cells, VEGF-A promotes muscle regeneration through transdifferentiation or fusion of these cells, although there is evidence to refute this mechanism. For a long time, VEGF-A was considered an endothelial-specific growth factor that promotes a strong angiogenic response. However, current studies demonstrate the ability of VEGF-A to induce myofibril regeneration in skeletal muscle. In addition to angiogenesis, the interaction of VEGF-A with its receptors is important for the survival and stimulation of cell differentiation in a wide range of different tissues, including skeletal muscle [8].

##### VEGF and “Angiogenic Switch” in the Myocardium

Studies by many authors have shown that physical activity has a positive effect on the coronary vasculature, including myocardial oxygen demand, endothelial function, autonomic tone, markers of inflammation, and the development of collateral coronary vessels [11,12]. However, the direct effect of exercise on myocardial angiogenesis remains poorly understood. Physical activity is accompanied by functional, electrical, and structural remodeling of the heart. Myocardial hypertrophy is accompanied by an increase in the left atrium diameter, the left ventricle wall thickness, and myocardial mass. Vascular changes in the myocardium are reported to be associated with neoangiogenesis and capillary density in the heart muscle [13]. Angiogenesis in the myocardium is a complex process mediated by the interaction of proangiogenic and anti-angiogenic factors. Cardiomyocytes express VEGFR-1 and VEGFR-2, which are rapidly activated in response to hypoxia [14]. It is believed that the role of VEGF-A in myocardial remodeling is related to the balance of its actions on VEGFR-1, which inhibits the development of hypertrophy, and VEGFR-2, which has prohypertrophic effects. A transgenic murine model of VEGFR-1 deletion of endothelial cells led to angiogenesis and the development of cardiomyocyte hypertrophy through signaling of erb-B tyrosine kinase [15]. These processes can be further influenced by microRNAs (miRNAs) such as miR-374, which is known to positively regulate cardiomyocyte hypertrophy in murine models. Vascular endothelial growth factor-B (VEGF-B) and angiopoietin 1 (ANGPT-1) are the main angiogenic factors that enhance endothelial cell proliferation and accelerate vascular growth in the infarction zone [13]. Among the various types of mechanisms that regulate gene expression, epigenetic changes associated with the acetylation–deacetylation of histones by various isoforms of histone deacetylase (HDAC) are also important [16]. Recent research indicates that changes in oxidant/antioxidant balance can affect angiogenic activity in tissues [17]. These oxidative changes are the result of reactive oxygen species (ROS). ROS can change the structure of proteins, lipids, carbohydrates, and nucleic acids. According to the authors, damage to the mitochondrial membrane is realized through ROS radicals derived from xanthine oxidase of inflammatory cells [ibid.]. Alternatively, during oxidation, the high rate of destruction of red blood cells from intense endurance exercise can increase the amount of free iron, which will help protect highly reactive hydroxyl radicals through iron-catalyzed Haber–Weiss reactions. Ardakanizade et al. [13] studied short-term and long-term adaptive changes occurring in the myocardium as a result of physical exertion on endurance to elucidate the molecular picture of tissue remodeling. In addition, the authors tried to reveal the effect of an exercise regimen on oxidative changes and angiogenesis in the heart muscle. Experimental studies conducted in animals showed high myocardial plasticity as a result of endurance exercises, which was expressed by changes in the signaling cascade at the level of transcription and translation. These changes increased the contractile ability of the myocardium, and consequently, the cardiovascular system potential [18]. A significant increase in myocardial contractility was accompanied by a significant increase in its mass and left ventricle wall thickness. The study reported the presence of the heart muscles impairment, which was accompanied by oxidative changes. The results showed that oxidative stress was dependent on the duration of endurance exercise. The decrease in antioxidant capacity was more pronounced with prolonged exercise, indicating an increase in oxidative changes caused by the duration of the exercise program. Oxidative changes have been expressed as an increase in oxidative markers (nitric oxide (NO)) and the end product of lipid peroxidation (MDA). With prolonged endurance exercise, vasodilation occurs due to the need for blood supply to the heart muscle. Therefore, NO is involved in blood supply, helping coronary arteries through endothelial nitric oxide synthase (eNOS) [19]. This blood supply is always accompanied by an increase in NO and the expression of the gene for vascular endothelial growth factor beta (VEGF-B) in the molecular structure.

*VEGF-B* becomes active due to the interaction between a hypoxia-inducible factor-1 (HIF-1) and a hypoxia reactive element (HRE) in response to hypoxia induced by prolonged endurance exercise [20,21]. *VEGF-B* stimulates endothelial cell proliferation as well as migration and new vessel formation through transmission signal carried out by tyrosine kinase receptors [22]. Thus, the need for blood supply to the coronary muscles is satisfied by the process of angiogenesis and expression of *VEGF-B*. As the expression of *VEGF-B* increased in the experimental group (performing endurance exercises for a long time), the same group demonstrated a higher level of angiogenesis. Thus, the higher production of ROS, which occurs concurrently with the higher expression of *VEGF-B*, confirms the relationship between angiogenesis and oxidative stress. Exercise induces muscle tissue calcineurin signaling pathways to enable muscle tissue contraction. It has been shown in the literature that the regulation of calcineurin-dependent genes is mediated by the MEF2 transcription factors [23]. Moreover, it was indicated that the expression of the *MEF-2c* gene can be increased in endothelial cells via VEGF-B, which, in turn, regulates the process of vasculogenesis [24]. Therefore, a significantly higher expression of the *VEGF-B* gene and the *VEGF-B* gene induced by endurance exercise is associated with an increase in cardiac muscle contractility and vascularization. Angiopoietin 1 (ANGPT-1) is a protein that supports the survival of endothelial cells during angiogenesis. VEGF-B-induced vascular permeability can be blocked and regulated by overexpression of *ANGPT-1* [24]. Thus, the density of new vessels can be adjusted. Accordingly, a higher expression of *ANGPT-1* in the experimental group (where endurance exercises were performed for a short period of time) contributed to the angiogenesis regulation. However, this was not observed in another study group with a longer period of endurance exercises [13]. This suggests that with prolonged aerobic loads, regulation of angiogenesis is impaired and new blood vessels are formed. These data are consistent with the results of other studies, which reported that concomitant expression of VEGF-B and angiopoietin-2 can increase and regulate the density and permeability of microvessels [25].

After birth, mammalian cardiomyocytes become differentiated and generally lose their ability to proliferate. However, a certain level of their renewal occurs throughout life [26]. Note that in adulthood, the growth of the heart occurs more due to hypertrophy of cardiomyocytes than due to hyperplasia. Myocardial hypertrophy can be either physiological or pathological. Physiological hypertrophy is the result of a response to exercise or other stimuli. Normally, a hypertrophied heart exhibits improved vascular perfusion and metabolism, and the growth process is initiated by molecular pathways specific for physiological hypertrophy [27]. Cardiac perfusion is largely controlled by the microvessel through a dense capillary network with approximately one capillary per muscle fiber. Consequently, being critical for neoangiogenesis, VEGF plays a central role in cardiomyocyte hypertrophy.

##### VEGF and “Angiogenic Switch” in Cerebrovascular and Cardiovascular Systems

The role of VEGF-A in maintaining the “angiogenic switch” balance in the cardiovascular and cerebrovascular systems is beyond doubt. The positive effects of VEGF-A are associated with the protection of endothelial cells by increasing the expression level of antiapoptotic proteins and NO synthesis [28]. VEGF causes severe endothelium-dependent vasodilation, primarily through nitric oxide (NO) and prostacyclin (PGI2). In cultured human endothelial cells, binding of VEGF-A to VEGFR-2 activates the PI3K/Akt pathway, followed by activation of endothelial NO synthase (eNOS) and NO release. However, VEGF-induced vasodilation is not reversed by inhibition of NO and PGI2, suggesting that an endothelial-derived hyperpolarizing factor (EDHF) is also involved in VEGF-mediated vasodilation [29]. The negative effects of VEGF-A have been reported in several studies. The ability of VEGF-A to induce atherogenesis and its effect on endothelial cells as a mitogen through re-endothelialization were reviewed by Braile et al. [30]. The mitogenic effect of VEGF-A on endothelial cells is mediated through VEGFR-2 and the activation of kinase is regulated by extracellular signals (ERK1/2). Phosphorylation of VEGFR-2 leads to the activation of phospholipase C (PLC-γ), which in turn stimulates the Raf-MAPK/ERK (MEK)-ERK1/2 kinase cascade in cultured endothelial cells [29]. VEGF-A plays a role in cell migration, including vascular smooth muscle cells, monocytes, and polymorphonuclear cells. In endothelial cells, cell migration is induced by activation of focal adhesion kinase (FAK) and paxillin, as well as via the phosphatidylinositol kinase (PI3K)/Akt and MAPK pathways [29]. The ability of VEGF-A to induce adhesion of monocytes through transendothelial migration and activation [31], improving endothelial permeability and increasing expression of the adhesion protein and chemoattractant protein-1 of monocytes has also been described [32]. VEGF-A was previously known as a vascular permeability factor. Its interaction with VEGFR-2 on cultured endothelial cells triggers the activation of several pathways that regulate the adhesive properties of the transmembrane protein, vascular endothelial cadherin (VE-cadherin). These pathways include the protooncogene tyrosine kinase src and culminate in phosphorylation and internalization of VE-cadherin, separating it from the cytoskeleton. This subsequent loss of adhesive bond results in a marked increase in vascular permeability.

Ylä-Herttuala et al. found no VEGF-A or its receptors in intact segments of human coronary vessels. However, their expression increases in endothelial microcapillaries, in macrophages, and in partially differentiated smooth muscle cells of atherosclerotic lesions [33]. In their research on animal model of rabbits exposed to 8 weeks of chronic stress, Yu et al. revealed instability of atherosclerotic plaques and activation of angiogenesis caused by the release of VEGF-A which was found in large amounts in blood serum [34].

##### VEGF and “Angiogenic Switch” in Lung Tissue

Sports loads in cyclic sports are associated with an increase in oxygen consumption. That is why the pulmonary system becomes the main system subject to functional changes during exercise. Numerous studies of the sports physiology have demonstrated a clear relationship between heart rate, oxygen consumption, respiratory rate and minute ventilation, energy metabolism, and lactate production [35]. Although the lungs are one of the limiting systems of the body in cyclic sports, their response to high-intensity loads and other environmental stresses is often overlooked. Vigorous exercise can contribute to capillary leakage, especially when left atrial pressure rises due to left ventricular (LV) systolic or diastolic insufficiency. LV diastolic dysfunction, which causes an increased pressure in the left atrium during exercise, often results in pulmonary edema and capillary hemorrhage. Studies suggest that the lungs can respond to exercise and immersion stress with pulmonary edema and pulmonary hemorrhage [36].

There are many controversial questions about the role of VEGF-A in the physiology and pathology of the lungs. Lung tissue contains the highest level of transcripts among the wide range of organs expressing VEGF-A. The lung is one of the main organs in which VEGF-A controls several important physiological functions [37]. Lung morphogenesis requires constant physical and molecular interactions between the mesenchymal stroma and epithelial elements. Airway epithelial cells are the predominant source of VEGF-A throughout lung organogenesis and appear to be critical for normal alveolarization, rapid alveolar proliferation during lung maturation, and normal vascular development. It is believed that VEGF-B, VEGF-C, VEGF-D, and PlGF also play a role in the physiological development of the lungs [38], but they are not extensively studied in this context or not so extensively as VEGF-A. There exists much research on these other growth factors. During exercise and airway activation, the lungs gradually receive a rich blood supply through the growth of endothelial cells and vascular cells in the pulmonary mesenchyme. This growth is accompanied by the expression of VEGF-A and its receptors, which play central morphogenetic functions in the lung tissue [39]. Pulmonary VEGF-A is synthesized by alveolar epithelial cells, bronchial epithelial cells, smooth muscle cells, and alveolar macrophages. This topographic compartmentalization allows VEGF-A to interact with components of the extracellular matrix, thereby creating concentration gradients that regulate the physiological functions of VEGF-A in the lungs [39]. Overexpression of pulmonary VEGF-A results in markedly dysmorphic lung structure. In contrast, neutralization of VEGF-A throughout fetal development by the extracellular domain Fc-VEGFR1 promotes a clearly simplified lung in neonatal mice [40,41]. Inhibition of VEGF-A leads to regression of tracheal capillaries and death of endothelial cells in the lungs of adult mice, while most vessels become resistant to VEGF-A cleavage after embryonic development [41]. It has recently been shown that this VEGF-A is an isoform of both VEGF-A xxx a and VEGF-A xxx b [42]. Similarly, VEGF-A receptors and coreceptors are also expressed by several types of cells in the normal lung on either side of the alveolar capillary membrane (ACM), including ATII cells. The classical processes associated with VEGF-A activity (permeability, angiogenesis, and mitogenesis) are extremely limited in the mature lung. Thus, although the exact role of VEGF-A in the lungs is not fully defined, it has been suggested that compartmentalization of VEGF-A in the alveolar space of intact lung tissue is essential to maintain normal lung structure and function.

In addition to its well-known functions as a trophic factor and growth factor, VEGF-A may play a new biological role in maintaining lung homeostasis. Cellular homeostasis of the lung tissue requires the rapid removal of apoptotic cells in order for their total number to remain constant. Effective removal of damaged cells reduces the risk of necrosis and inflammation, and by binding of apoptotic cells to the phosphatidylserine receptor, many immunosuppressive cytokines (TGF-β, PGE2, PGI2, and IL10) are released to suppress inflammation and reduce the risk of autoimmune diseases [43]. VEGF can promote efferocytosis, which, in turn, leads to further VEGF-A production and cell repair during lung tissue damage [44].

Chin-Kuo Lin et al. studied the expression patterns of inflammatory cytokines and growth factors after pulmonary hyperventilation (VILI) and determined the phenotypes of monocytes recruited to the lungs during recovery to elucidate how monocytes recruited to the lungs and pulmonary VEGF-A promote epithelial proliferation [45]. The increased expression of VEGF-A and TGF-β found by the authors in injured lungs underlines the role that VEGF-A and TGF-β play in lung recovery after VILI. Proteins belonging to the families of epidermal growth factor and fibroblast growth factor are involved in the restoration of damaged lung epithelium [46]. In addition to its well-known angiogenetic properties, VEGF-A acts as a powerful epithelial lung mitogen involved in repairing lung damage [47,48,49]. Studies have shown that the effects of VEGF-A vary depending on the damaged cells in the lung tissue and the recovery time [38,50,51]. VEGF-A receptor signaling is required for the maintenance of alveolar structures in normal rat lungs, as well as for the regulation of proliferation and apoptosis of damaged alveolar epithelial cells in an autocrine or paracrine manner [52]. Acute respiratory distress syndrome (ARDS) is the most severe form of lung disease. Medford et al. found that VEGF-A expression was significantly increased in late ARDS cases after day 7 compared with controls (without lung tissue damage) and early ARDS cases within 48 h [53]. Chin-Kuo Lin et al. [45] analyzed VEGF-A expression and the number of Ki67-positive cells in the alveolar epithelium of lung slices and found a strong positive correlation between VEGF-A expression and alveolar epithelial cell proliferation during VILI recovery. Their results demonstrated that VEGF-A can help repair alveoli in damaged lungs during healing [45]. Taken together, these findings suggest a dual role for VEGF-A in lung tissue repair: Increased serum VEGF-A concentration promotes progressive lung damage early in the disease, while increased VEGF-A expression in lung tissue promotes lung recovery during the late recovery phase. Thus, the revealed information about positive and negative effects of VEGF-A indicates that while considering the therapeutic effect of VEGF-A on lung tissue under intense physical activity the biology of VEGF-A in the lungs should be taken into account.

#### 3.1.2. Association of the VEGF Gene SNVs with Changes in the “Angiogenic Switch” Stroke and Functional Resources in Athletes

**Figure 1:**
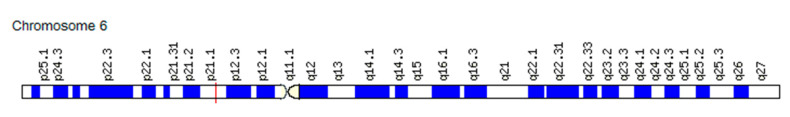
Localization of the VEGF gene on chromosome 6p21.1.

The *VEGF* gene encoding the homonymic protein plays a key role in the regulation of vasculogenesis and angiogenesis. It was identified, isolated, and cloned more than 25 years ago [54]. It is localized on chromosome 6p21.1 (Figure 1).

There are several related genes, including *VEGF-B* and *VEGF-C,* but the greatest attention is paid to *VEGF-A* because of its key role in the regulation of angiogenesis, in both physiological and pathological homeostasis. All isoforms of the VEGF protein have specific receptors. They have different affinities for the different receptors and can bind to them. VEGF-B overrides the activity of VEGF-A by activating VEGFR-1. VEGF-C and VEGF-D can be angiogenic factors. In this case, they are activated via the VEGFR-2 and VEGFR-3 receptors. Furthermore, these factors can be lymphangiogenic (mainly VEGF-D) and are activated through the VEGFR-3 receptor. The absolute and relative levels of expression of VEGFR-2 and VEGFR-3 in the endothelium can influence the nature of the effect of growth factors VEGF-C/D—angiogenic or lymphangiogenic effects. *VEGF* expression is stimulated by a variety of proangiogenic factors, including hypoxia-induced factor (HIF), epidermal (EGF), and fibroblast (FGF) growth factors. In addition, the blood pH, the partial pressure, and the O_2_ concentration in the inhaled air affect the VEGF level. Although VEGF primarily targets endothelial cells, it has been shown to have multiple effects on additional cell types. Primarily, VEGF-mediated pathogenic effects are caused by its effect on vascular permeability and neoangiogenesis (neovascularization) [54].

The severity of expression of genes encoding the process of angiogenesis is reported to be associated with the intensity of physical activity [55]. This confirms the point of view, according to which the change in the “angiogenic switch” stroke is a marker of athletes’ physical performance. Among the studied single nucleotide variants (SNVs) of *VEGF*, SNVs in the promoter (regulatory) region are of particular interest. For example, the replacement of cytosine with guanine at position −634 (−634 G/C; rs2010963) increases the activity of the gene and, accordingly, determines individual differences in the level of its expression [56].

Akhmetov et al. [5] investigated the frequency distribution of VEGF-A alleles in athletes involved in cyclic sports and in a control group of subjects not involved in sports. The authors also evaluated the association of the studied SNV *rs2010963* (−634 G/C) genotypes of *VEGF* with the aerobic performance of athletes and the control group. The frequency of the C allele in the control group was 25.3% in women and 23.6% in men (*p* ≤ 0.05). The distribution of genotypes in the control group was as follows: GG—57.6%; GC—35.8%; CC—6.6%. The frequency of the C allele was statistically significantly higher in the group of athletes than in the control group (29.2% versus 24.5%, respectively; *p* = 0.0026). The distribution of genotypes among athletes was as follows: GG—50.6%; GC—40.4%; CC—9%. Analysis of the distribution of alleles in men and women did not reveal statistically significant differences in both groups (*p* ≥ 0.05). A higher frequency of the C allele was found in long-distance runners compared to athletes in other sports. This indicates that the C allele can be a genetic predictor of the development and endurance manifestation in athletes.

Considering that SNVs of the *VEGF* gene promoter can alter the expression of the *VEGF* gene and the level of the VEGF-A protein in tissues, Prior et al. [57] suggested that SNVs −2578/−1154/−634 in the promoter region of the *VEGF* gene are associated with the expression of *VEGF* in human myoblasts and maximal oxygen consumption (MOC) before and after aerobic exercise. The authors analyzed the effect of the *VEGF* promoter region haplotype −2578/−1154/−634 on the *VEGF* expression using the luciferase reporter assay in cultured human myoblasts. In a study with exposure to hypoxia, it was found that haplotypes CGG and AGG showed the lowest hypoxic induction (1.5 and 2.0 times, respectively), while haplotypes AAG and CGC showed the highest hypoxic induction of the *VEGF* gene expression (3.1 and 3.2 times, respectively). According to the authors, the results obtained demonstrated the potential functional effect of SNVs −2578, −1154, and −634 as follows: the combination of G-alleles (AGG and CGG haplotypes) leads to a decrease in the *VEGF* gene expression in cultured human myoblasts compared to the AAG and CGC haplotypes; the presence of A or C alleles in −2578 SNV (the first position in the haplotype) did not statistically significantly affect the expression of the *VEGF* gene. The data obtained showed that the influence of these haplotypes corresponded to the dominant/recessive genetic model, given that only subjects with two copies of AGG or CGG haplotypes showed low BMD. Subjects with ≥1 copy of AAG or CGC haplotypes showed higher BMD values. As none of these three SNVs has been found at any particular transcription factor binding site identified to date, the exact mechanism of the effects of the VEGF −2578/−1154/−634 haplotype on the *VEGF* gene expression remains unknown. It is possible that these SNVs disrupt binding sites for transcription factors that are not yet identified or affect interactions between transcription factors. For example, the hypoxia response element (HRE) in the promoter region of the *VEGF* gene (5′-position from −2012 to −2005) requires interaction with the upstream protein activator−1 (5′-position from −2166 to −2160) and the downstream protein activator −2α (5′-position from −1117 to −1110), which lie within the test sequence in the promoter region. The authors suggested that SNVs −1154 and/or −634 might somehow influence these interactions [57].

Studies conducted by Arsic et al. [8] used immunohistochemical analysis to show that in intact muscle fibers neither VEGFR-1 nor VEGFR-2 is expressed. In contrast, damage to muscle fibers led to a marked increase in the presence of these receptors. In particular, both receptors were strongly expressed by elongated cells surrounding the newly formed fibers, which could be identified by the presence of a central nucleus resembling the appearance of activated satellite cells at the edge of regenerating fibers. Expression was detected early after injury and persisted until later stages of the regenerative process. In addition, highly expressed VEGFR-2 was found on the surface of mature muscle fibers in the early recovery period after injury. Most strikingly, overexpression of PlGF, a VEGFR-1 agonist, did not promote muscle regeneration after injury, even at very high doses of the vector. These results clearly indicate that VEGFR-2 is a major mediator of VEGF action on myogenic cells.

Currently, at least two signaling pathways are known that are important for muscle regeneration. The first pathway is the activation of VEGFR-2 in endothelial cells, namely, PI3K/Akt. The second is kinase pathways that lead to increased expression and activity of the MyoD protein. Activation of Akt signaling in muscle cells is important for the suppression of apoptosis during differentiation and growth of myofibrils [58]. Interestingly, insulin growth factor-1, a powerful promoter of muscle regeneration that stimulates muscle differentiation through Akt, also increases VEGF synthesis in cells, indirectly suggesting the involvement of VEGF in the regeneration process [59]. Accordingly, muscle fibers transduced with active Akt also produce increased levels of VEGF and show signs of muscle hypertrophy. Thus, the SNV rs2010963 (−634 G/C) of the *VEGF* gene is associated with the physical performance of athletes and plays a key role in sports selection. The role of other studied SNVs needs to be clarified. The results of these studies are of both fundamental and applied importance as they contribute to a better understanding of the molecular adaptation mechanisms of the cerebrovascular and cardiovascular systems to aerobic loads as well as facilitate the choice of optimal sports specialization and type of professional training of athletes.

### 3.2. Fibroblast Growth Factor (FGF)

#### 3.2.1. The Role of FGF in Angiogenesis in Athletes

Fibroblast growth factor (FGF) was originally identified as a protein capable of stimulating fibroblast proliferation. It is currently known to have 22 isoforms, although there are only 18 receptors for them (FGFR). Four FGF isoforms (FGF11, FGF12, FGF13, and FGF14) do not bind to FGFR [60]. FGFs perform multiple functions through FGFR binding and activation. Activated FGFRs mediate signaling by recruiting specific molecules that bind to phosphorylated tyrosine in the cytosolic portion of the receptor, triggering a number of signaling pathways leading to specific cellular responses. They then serve as docking sites, docking proteins, or signaling enzymes. Signaling complexes are formed and recruited to active receptors, leading to a cascade of phosphorylation events [61]. The main signaling pathway for stimulating FGFRs is the RAS/MAP kinase pathway, and the PI3/AKT kinase pathway and the PLCγ pathway have also been studied. Unlike other growth factors, FGF works with heparin or heparan sulfate proteoglycan (HSPG) to activate FGFR and induce pleiotropic responses that lead to a variety of cellular responses [62]. Expression of FGF-binding proteins (FGFBP) can modulate FGF-dependent vascular permeability and is considered to be an “angiogenic switch” in the regeneration of many tissues, including skeletal muscle, tendons, myocardium, blood vessels, lung, and nervous tissue [60]. The physiological role of FGFs as an “angiogenic switch” depends on the FGF signaling pathway. This interaction results in new sites for a set of proteins that are responsible for the activation or weakening of signaling [30]. Four isoforms of FGF (FGF1, FGF2, FGF4, and FGF6) are the most studied as proangiogenic factors.

##### FGF and the “Angiogenic Switch” in Skeletal Muscle

Skeletal muscle regeneration is largely controlled by FGFs, which are abundant in the regenerating muscle regions. Of greatest interest is the FGF6 isoform, as it is most specific for muscles and is strongly activated upon injury [63].

Yablonka-Reuveni et al. studied the role of FGF in recruiting skeletal muscle satellite cells. They demonstrated the high efficiency of the FGF2 isoform in stimulating the proliferation of satellite cells [64]. Doukas et al. investigated the activity of FGF isoforms in skeletal muscle recovery. In particular, plasmid and adenoviral vectors were immobilized in a collagen–gelatin mixture, which was then delivered to damaged muscle tissue. The results demonstrated an angiogenic response in muscles (microvascular development) in the early stages of injury. Subsequently, the authors discovered arteriogenesis or the development of higher-order vessels, which contained medial layers of smooth muscle cells, as well as increased muscle tube regeneration after delivery of FGF2 or FGF6 isoforms to damaged muscle tissue [65,66].

Richardson R.S. studied the expression of angiogenic growth factors in skeletal muscles in response to a single physical exercise (in particular, b-FGF and a-FGF in the gastrocnemius muscles) [67]. Animal models were injected with b-FGF and a-FGF intramuscularly into the right gastrocnemius muscle. Exercise significantly reduced vascularization in both the right and left calf muscles compared to control (without exercise), probably because of changes in muscle mass. Intramuscular injection of b-FGF significantly increased vascularization locally in the right muscle into which it was injected, while intramuscular injection of a-FGF did not lead to vascularization of the right muscle. With the simultaneous administration of angiogenic factors and physical activity, only b-FGF caused a significant local increase in vascular growth in the muscle under study. Neither b-FGF nor a-FGF elicited any angiogenic action on the left side. This can be explained by the one-sided effect of exercise.

##### FGF and “Angiogenic Switch” in the Myocardium

Left ventricular hypertrophy is an adaptive response of the heart to exercise, but it is also a risk factor for cardiovascular mortality. Among the factors contributing to the transition from adaptive to pathological remodeling of the heart, an imbalance of the “angiogenic switch”, leading to the development of defects in cardiac angiogenesis and vascularization, plays a significant role [14]. Changes in hemodynamic and mechanical factors, as well as hypoxia, leading to a mismatch between the oxygen demand of the heart and its blood supply, stimulate the release of angiogenic growth factors from cardiomyocytes in order to cause a parallel growth of the feeding vasculature.

Major fibroblast growth factor (bFGF, also called FGF2) is also expressed in endothelial cells, including the heart, and has been shown to enhance the development of vascular collaterals in the myocardium in an animal model of coronary occlusion in dogs [68]. Rajanayagam et al. established the proangiogenic role of FGF2 in canine ischemic myocardium [69].

Alternatively, the role of FGF2 in myocardial vascularization during hypoxia caused by pressure overload is less well understood. For example, thyroxine, a potent stimulator of cardiac hypertrophy and vascularization, has been shown to enhance FGF2 expression and increase cardiac capillary endothelial cell proliferation and angiogenesis [70].

Santiago et al. investigated the differential effects of high and low molecular weight FGF2 isoforms on myocardial hypertrophy, fibrosis, and inflammation [71]. Echocardiographic measurements, gravimetry, and cross-sectional area of cardiomyocytes showed that the absence of FGF2 results in a statistically less degree of hypertrophy during pressure overload. The presented results demonstrate that FGF2 is the main stimulating component of the growth of myocardial hypertrophy. In addition, it was shown that hemodynamic stress, rather than FGF2 and myocardial hypertrophy, correlates with switching of the studied isoforms. Despite the fact that transcriptional and post-transcriptional control determine the ratios of myosin isoforms in cardiomyofibrils, the isoform switching that occurs during hemodynamic stress is the result of changes in transcriptional rather than posttranscriptional regulation.

Yajima et al. evaluated whether FGF2 and/or heparin, which induce angiogenesis, affect myocardial function in hypertensive conditions [72]. The study showed that intramyocardial injection of FGF2 (plus heparin) in rats with hypertensive myocardial hypertrophy was associated with significant improvements in systolic pumping function and ventricular dilatation, as well as an increase in myocardial capillary density.

Chen et al. investigated other isoforms of FGF, such as acidic fibroblast growth factor (sp-FGF1) or FGF1 [73]. It has been found that a secreted version of the *FGF1* gene contributes to the functional improvement of disrupted endothelial progenitor cells and that autologous transplantation of modified sp-FGF1 in these cells may promote neovascularization in a porcine model of chronic myocardial ischemia.

To elucidate the pathophysiological role of FGF in cardiomyopathy, Tomita et al. evaluated myocardial biopsies from 24 patients (nine with hypertrophic cardiomyopathy, 12 with dilated cardiomyopathy, and three with hypertensive hypertrophy) and six controls. All samples were stained for FGF1 (also known as aFGF) and basic FGF (bFGF) by immunohistochemistry. FGF expression was significantly increased in cardiomyocytes obtained from the left ventricle of patients with cardiomyopathy. As it was revealed, FGF-1 can promote myocardial hypertrophy as a reparative response to myocardial damage in patients with idiopathic cardiomyopathy [74].

In recent years, a significant amount of knowledge has been accumulated on the regulation of the formation of new vessels in a hypertrophied heart [14]. It became clear that myocardial endothelial cells not only respond to hemodynamic forces and paracrine signals from neighboring cells, but also actively participate in the processes of cardiac remodeling, stimulating the growth and contractility of cardiomyocytes or the production of extracellular matrix proteins in myofibroblasts. Moreover, in response to adequate signals, they can change their phenotype and transdifferentiate into extracellular cells that produce matrix. As myocardial vascularization plays a central role in the transition from adaptive cardiac hypertrophy to heart failure, endothelial cells and signaling mechanisms involved in the regulation or dysregulation of angiogenesis in the myocardium represent promising therapeutic targets for improving cardiac remodeling caused by pressure overload and preventing the transition to heart failure, which is important in sports practice.

##### FGF and the “Angiogenic Switch” in the Cerebrovascular and Cardiovascular Systems

FGFs constitute one of the most versatile and complex families of signaling in vertebrates, playing critical roles in a wide variety of biological processes, including a wide range of vascular functions. The multiple isoforms of FGFs generated by alternative splicing may play different roles in vascular development. FGFs are broad-spectrum mitogens that stimulate various cellular functions, including migration, proliferation, and differentiation [75]. The expression pattern of FGFs is highly variable, from almost ubiquitous (FGF1 and FGF2) to highly restricted, to certain cell subpopulations at certain stages of development (FGF3, FGF4, FGF8, FGF17, and FGF19). In pathological conditions such as inflammation, etc., FGF is secreted in large amounts by various types of cells, including monocytes, tissue macrophages, endothelial cells, stromal cells, and tumor cells [76].

Despite the recognition of FGF as a strong proangiogenic factor, the deciphering of its exact functions in the vascular system has not been adequately studied [77]. Mutations in the Fgfr1 or Fgfr2 genes in mouse embryos led to embryonic lethality at very early stages of development, which makes it impossible to further assess their contribution to vascular development [78]. By contrast, studies of angiogenic FGF isoforms such as FGF1 and FGF2 did not reveal abnormalities in embryonic vascular development, implying that there is significant redundancy in the system of these isoforms [79]. One of the difficulties in investigating the FGF system arises from its erratic action on various types of cells and tissues. However, using tissue-specific promoters, recent research has begun to uncover the role of FGFs in the “angiogenic switch” in the cardiovascular and cerebrovascular systems.

Analysis of FGF signaling in the vasculature of adult mice revealed a significant contribution of FGF to vascular development as well as maintaining vascular integrity. In adult mice, FGF is required for basal endothelial signaling and maintenance of vascular homeostasis. Inhibition of signaling through FGF leads to disassembly of endothelial junctions, progressing to severe disruption of vascular integrity [80]. In contrast to VEGF, which causes degradation of VE-cadherin-based compounds through activation of Src, FGF enhances the adhesion of the compounds by enhancing the binding of VE-cadherin to p120 catenin. The critical role of FGF signaling in neovascularization is also demonstrated in a more recent study that describes the cross-signaling mechanism between FGF and VEGF. The *VEGFR2* gene expression levels are tightly controlled by endothelial FGF signaling, which is capable of activating VEGFR2 transcription through an Ets-dependent manner. Thus, FGF indirectly promotes neovascularization by regulating endothelial sensitivity to VEGF [81]. It has been repeatedly shown that although FGF-induced neovascularization is often impaired by VEGF inhibition in various angiogenic models, VEGF-induced vascular formation is not so strongly affected by depletion of FGF signaling [82]. Together, these studies support the hierarchical control of new vessel formation by which the FGF system promotes new vessel growth by controlling VEGF signaling.

##### FGF and the “Angiogenic Switch” in Lung Tissue

Angiogenesis is a central component of the pathophysiology of lung tissue in various chronic diseases. The “angiogenic switch” controls the progression of airway remodeling using a variety of pro- and anti-angiogenic factors, including FGF. FGF signaling plays an important role in the development, homeostasis, and regeneration of lung tissue. Of all the FGF isoforms in the angiogenesis of the lung tissue, bFGF makes the greatest contribution [83]. Unlike VEGF, bFGF requires basement membrane proteolysis or cell damage in order for it to be released and bind to a variety of cellular targets. Rabata et al. using three-dimensional models of mouse lung tissue cell cultures, studied the role of FGF ligands and the interaction of FGF signaling with epithelial growth factor (EGF) in the morphogenesis and differentiation of lung epithelium [84]. In the absence of adhesion, FGF signaling promoted the formation of lung spheres from epithelial stem/lung progenitor cells (LSPCs). Ultrastructural and immunohistochemical analyzes have shown that LSPCs produce more differentiated progeny lung cells. In a three-dimensional extracellular matrix, FGF2, FGF7, FGF9, and FGF10 promoted the formation of organelles in the lung tissue, and FGF9 showed a reduced ability to stimulate the formation of organelles in the lung tissue. The authors hypothesized that FGF9 has a reduced ability to maintain LSPC survival and/or initial differentiation. FGF7 and FGF10 produced larger organelles and induced organoid branching at a higher frequency than FGF2 or FGF9. The higher concentration of FGF2 increased the efficiency of organelle and pulmonary formation in the lungs. In this regard, the authors concluded that the level of FGF2 signaling is a decisive factor in the survival and differentiation of LSPC, as well as morphogenesis of the lung epithelium. The studied FGF isoforms showed differences in the stimulation of different types of lung epithelial cells. FGF9 was a potent inducer of more proximal cell types, including ciliated and basal cells. FGF7 and FGF10 directed differentiation to distal lung clones. The WNT signaling pathway increased the efficiency of lung organoid formation, but in the absence of FGF10 signaling, organelles exhibited limited branching and a less differentiated phenotype [84].

#### 3.2.2. Association of FGF Gene SNVs with Changes in the “Angiogenic Switch” Stroke and Functional Resources in Athletes

The mammalian Fibroblast Growth Factor (FGF) family contains 22 genes, eight of which encode molecules that transmit a signal through the receptors of FGF tyrosine kinase. Secreted signaling FGFs can be grouped into subfamilies based on biochemical function, sequence similarity, and evolutionary relationships. Currently, there are five subfamilies of paracrine FGFs, one subfamily of endocrine FGFs, and one subfamily of intracellular FGFs [85]. Members of the FGF family are involved in a variety of biological processes, including embryonic development, cell growth, morphogenesis, organogenesis, tissue repair, tumor growth, and invasion, and have broad mitogenic and cell survival activities. FGF functions as a modifier of endothelial cell migration and proliferation, as well as an angiogenic factor. There are very few studies on the association of *FGF* gene SNVs with functional resources of the body. This may be because disturbances in the *Fgfr1* or *Fgfr2* phenotypes in embryos of animal models lead to embryonic lethality at very early stages of development, which makes it impossible to further assess their contribution to the development of organs and systems of the body. Among the studied database, there are sporadic descriptions of clinical cases of rare heterozygous deletions of copy number variants or SNVs with the participation of FGF. In the studies of Karolak J.A. et al., a complex inheritance of lethal developmental disorders of the lungs due to disruption of the TBX-FGF pathway has been described [86]. The emergence of rare coding variants involving FGF10 with putative hypomorphic noncoding SNVs implies a complex inheritance of pulmonary hypoplasias. Moreover, the existence of such rare coding variants supports the importance of TBX4-FGF10-FGFR2 epithelial–mesenchymal signaling in human lung organogenesis and helps explain the histopathological continuum observed in these rare lethal disorders of lung development. Individuals with coding variants including either TBX4 or FGF10 also had at least one noncoding SNV in the predicted lung-specific enhancer region, which was absent in 13 control individuals with overlapping deletions but no structural lung abnormalities.

### 3.3. Platelet Growth Factor (PDGF)

#### 3.3.1. Role of PDGF in Angiogenesis in Athletes

Recent studies have demonstrated much evidence supporting the contribution of PDGF/PDGFR to the development of angiogenesis in both normal and pathological conditions [87]. PDGF is composed of A/B/C/D chains that are encoded by separate genes and are independently regulated. To date, five PDGF isoforms have been identified: PDGF-AA, -BB, -AB, -CC, and -DD [88]. These isoforms act through two receptor tyrosine kinases: PDGF receptors α and β. Isoforms PDGF-A and PDGF-B undergo intracellular activation during transport in the exocytic pathway for subsequent secretion, while isoforms PDGF-C and PDGF-D are secreted as latent factors that require activation by extracellular proteases. The polypeptide chains of PDGF-A and PDGF-B are well studied and regulate several physiological and pathophysiological processes, mainly using cells of mesenchymal or neuroectodermal origin as targets. The discovery of two additional ligands for two PDGF receptors suggests that cellular signaling mediated by PDGF is more complex than it was previously thought [89]. The binding of the ligand to the receptors causes dimerization of the receptor and leads to the activation of the tyrosine kinase domain and the subsequent recruitment of signaling proteins. The activation of these pathways causes different cellular responses.

##### PDGF and the “Angiogenic Switch” in Skeletal Muscle

High physical activity in sports places high demands on skeletal muscles and requires the delivery of a large amount of substrates and oxygen. This function is performed by blood vessels. Therefore, the formation of an integrated vascular network is a key factor in the growth and regeneration of skeletal muscles when they are damaged [90]. Analysis of recent publications has shown that, unlike VEGF and b-FGF, which are directly involved in the angiogenic process of skeletal muscles, PDGF is indirectly involved in this process. PDGF mainly stimulates the proliferation of cultured smooth muscle cells and cells of neuroectodermal origin that express the PDGF-b receptor. The role of isoforms of the PDGF family and their signaling pathways involved in exercise-induced skeletal muscle angiogenesis remains to be elucidated [90].

##### PDGF and “Angiogenic Switch” in the Myocardium

Exercise can cause an increase in myocardial mass by increasing the size of cardiomyocytes and/or the formation of new cardiomyocytes [91]. Cardiomyocytes are formed either from pre-existing cardiomyocytes or from cardiac progenitor/stem cells. At the same time, it was noted that exercise-induced cardiomyocyte renewal in mammals is mainly mediated by pre-existing cardiomyocytes [92]. Xiang et al. attempted to gain a deeper understanding of the molecular and cellular mechanisms of myocardial growth associated with intense physical activity [93]. Taking advantage of the dual immunofluorescent label for α- and β-platelet growth factor receptors (PDGFR), it was found that cardiac telocytes were significantly increased in exercise-induced myocardial hypertrophy. Telocytes are a recently identified special type of interstitial cell that is present in many tissues and organs, including the myocardium. The study showed that telocytes form in tandem with heart stem/progenitor cells in niches of heart stem cells, participating in myocardial regeneration and repair. The authors hypothesized that α and β PDGFR may be a molecular mechanism that helps control the activity of telocytes, myocardial stem/progenitor cells, cardiomyocytes, or endothelial cells. Thus, the increase in telocytes mediated by the possible activation of α- and β-PDGFR can lead to physiological hypertrophy of the myocardium in response to physical activity as well as contribute to the development of a favorable cardiac phenotype in athletes [93].

##### PDGF and the “Angiogenic Switch” in the Cerebrovascular and Cardiovascular Systems

Regulation of vascular barrier function is critical to tissue homeostasis. The maintenance of existing vessels and the formation of new ones require active cellular signaling. Signaling systems and their components involved in vascular balance play an important role in the physiological regulation of vascular integrity. The integrity of the vessels, in turn, is successfully achieved due to the coordinated action of growth factors and cytokines, which can alter the function of vascular cells, especially endothelial cells [94]. Signaling via PDGF in the vascular system is an important link in the pericyte–endothelial interaction [95]. PDGFs are the main mitogens for mesoderm-derived cells such as fibroblasts, pericytes, and smooth muscle cells, as well as for some cell populations of neuroectodermal origin [89].

In their study on an animal model, Hellström et al. found that perivascular mesenchymal cells expressing PDGFR *β* in a mouse embryo respond to PDGF-BB (PDGF-B homodimer) produced by angiogenic endothelium [96]. Therefore, paracrine PDGF signaling is required for the recruitment and proliferation of mural cells, as it has been demonstrated that PDGF-B expression is particularly high in the end cells of angiogenic vessels and in the endothelium of growing arteries [96].

Lindahl et al. indicate an essential role of PDGF signals in maintaining the integrity of the vascular barrier. In genetic studies of PDGF-B and PDGFR, *β* null mutant mice die perinatally, showing lethal hemorrhage and edema caused by loss of pericytes in microvessels [97].

The absence of pericytes in the capillaries increases their diameter and causes microaneurysms due to endothelial hyperplasia, which suggests that the coating of pericytes negatively controls the proliferation of endothelial cells. Detailed analysis of *β*-PDGFR-deficient mice revealed that the vascular instability observed in these mice is altered by systemic VEGF activation. The structure of the endothelial junction is slightly altered in PDGFR *β*-deficient mice, and this is explained by the effect of VEGF, as the onset of endothelial hyperplasia precedes an abnormality of the endothelial junction [98].

The initial induction of differentiation of pericytes from mesenchymal progenitors appears to be independent of PDGF signaling and is most likely mediated by other factors such as TGF-*β*. Pericyte populations in different tissues are affected to varying degrees by the loss of PDGF signaling in developing PDGFR *β*-/- embryos. Therefore, PDGF signaling is thought to be important in the subsequent maturation process in angiogenic vessels, where PDGF-BB released from endothelial cells controls pericyte migration and proliferation [96].

Currently, there are studies that indicate the importance of the interaction of endothelium and pericytes for the formation of the blood–brain barrier [97,98]. In studies on mice with defective PDGF signaling in the vasculature, a deficiency of pericytes was observed. It was found that the degree of coverage of the vascular wall with pericytes determines the degree of vascular permeability. Interestingly, in the vasculature of the central nervous system, the formation of tight junctions and the transport of endothelial vesicles due to transcytosis are critical regulators of cerebral vascular permeability, which increases due to a deficiency of pericytes.

##### PDGF and the “Angiogenic Switch” in Lung Tissue

The high density of capillaries in the lung tissue in athletes is probably associated with a high metabolic rate in the epithelium of the respiratory tract, which is very active in secretory processes. In fact, oxygen consumption by the airway epithelium is comparable to oxygen consumption by the liver and heart. In the normal airway, the maintenance of vascular homeostasis is the result of a complex interaction between numerous pro- and anti-angiogenic factors [99].

PDGF is expressed in the airways by a variety of cells: mast cells, eosinophils, and airway epithelial cells. Mast cells secrete PDGF, as well as other growth factors, including VEGF, bFGF, and others [ibid.]. Currently, the effect of three PDGF isoforms (PDGF-AA, -BB, and -AB) on human respiratory tract fibroblasts has been studied.

Human eosinophils express PDGF-B in both peripheral blood and inflamed airway tissue. In the studies of Warshamana et al., the expression of PDGF in bronchoalveolar epithelial cells of mice was revealed [100].

Investigating the role of PDGF in the mechanism of remodeling of the lungs and airways, Shimizu et al. found increased expression of PDGF by human lung epithelial cells [101].

Lewis et al. found that platelet-derived PDGF-BB significantly increased procollagen I expression of fibroblasts in patients with severe bronchial asthma compared with patients with mild/moderate asthma and the control group. In addition, the baseline expression of PDGFR-β by fibroblasts was significantly higher in patients with severe bronchial asthma compared with other groups [102].

In a study by Ingram et al., it was found that mitogenesis stimulated by the cytokine IL-13 is mediated by the secretion of PDGF-AA, thus activating the growth of myofibroblasts through an autocrine mechanism. It has also been demonstrated that IL-13 acts in synergy with IL-1β, enhancing this process by activating PDGFRα, suggesting a synergistic effect of these two interleukins on PDGF-AA/PDGFRα-dependent fibroblast proliferation [103].

In a review article by Kardas et al. describing the role of PDGF in airway remodeling, α-2-macroblobulin (A2M) is reported as one of the main factors influencing the regulation of PDGF [104]. An increase in the local concentration of PDGF on the cell surface increased the proliferation of fibroblasts.

Studies conclusively support the importance of PDGF as a mitogenic factor for human airway smooth muscle. Hirst et al. [105] studied the role of three PDGF isoforms (-AA, -AB, and -BB) in these cells. The study found that human respiratory tract cells express 5–6 times more PDGFRβ than PDGFRα in response to stimulation with PDGF isoforms. PDGF-BB and its receptor, PDGFRβ, turned out to be the most involved in the process of cell proliferation in comparison with the other two isoforms, PDGFRα.

Kardas et al. indicate the role of PDGF in two processes: enhancement of collagen synthesis by fibroblasts and a significant effect on the cells of the respiratory tract—an increase in the area and number of cells along with cell migration to the epithelium [104].

The above studies do not determine whether PDGF is in any way a systemic biomarker for lung tissue pathology. However, PDGF is believed to be more involved in local (autocrine and paracrine) interactions than in systemic ones.

#### 3.3.2. Association of the PDGF Gene SNVs with Changes in the “Angiogenic Switch” Stroke and Functional Resources in Athletes

**Figure 2:**
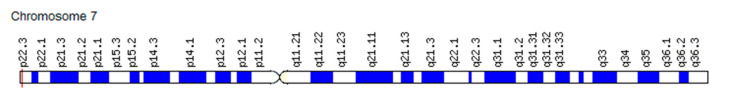
Localization of the PDGFA gene on chromosome 7p22.3.

The PDGF family consists of four polypeptide chains encoded by four different genes: *PDGFA*, *PDGFB*, *PDGFC*, and *PDGFD* [106]. The *PDGFA* (Platelet-Derived Growth Factor Alpha Chain) gene is located on chromosome 7p22.3 (Figure 2). This gene encodes a family of proteins that bind to and activate PDGF receptor tyrosine kinases (PDGFRs) that play an important role in a wide range of developmental processes.

**Figure 3:**
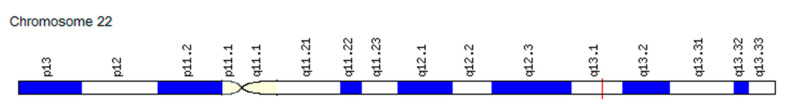
Localization of the PDGF-B gene on chromosome 22p13.1.

The *PDGF-B* gene (Platelet-Derived Growth Factor B Chain) is located on chromosome 22q13.1 (Figure 3). This growth factor plays an important role in the regulation of embryonic development, cell proliferation, cell migration, survival, and chemotaxis [106].

**Figure 4:**
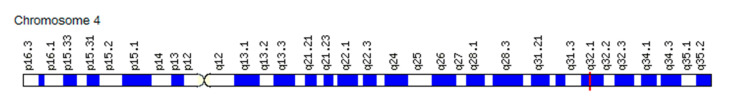
Localization of the PDGF-C gene on chromosome 4q32.1.

The *PDGF-C* (Platelet-Derived Growth Factor C) gene is located on chromosome 4q32.1 (Figure 4). The protein encoded by this gene is a member of the platelet growth factor family. It differs from the PDGF-A and PDGF-B polypeptides in the presence of an unusual N-terminal domain, the CUB domain. This protein plays an important role in the regulation of embryonic development, cell proliferation, cell migration, survival and chemotaxis, in angiogenesis, and blood vessel development. It participates in fibrotic processes. The CUB domain has mitogenic activity in smooth muscle cells of coronary arteries [106].

**Figure 5:**
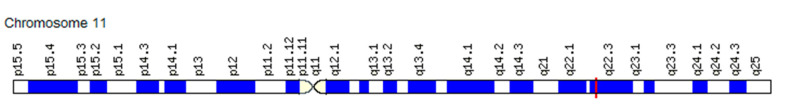
Localization of the PDGF-D gene on chromosome 11q22.3.

The *PDGF-D* (Platelet-Derived Growth Factor D) gene is located on chromosome 11q22.3 (Figure 5). This growth factor plays an important role in the regulation of embryonic development, cell proliferation, cell migration, survival and chemotaxis. It is a powerful mitogen for cells of mesenchymal origin. It plays an important role in wound healing. Moreover, it induces recruitment of macrophages, increased interstitial pressure, and maturation of blood vessels during angiogenesis [106].

All four proteins of the PDGF family have signal sequences of 18–22 amino acids in length with little homology between different types. The following portions, in both PDGF-A and PDGF-B, are ~60 amino acid propeptide sequences that are cleaved intracellularly from mature growth factors by furin or other proprotein convertases prior to secretion. PDGF-C and PDGF-D are proteolytically cleaved in the extracellular space [107]. The main domain of growth factor, which is actually the same domain as in VEGF, is followed by a highly polar tail in PDGF-A and PDGF-B [108]. PDGF receptors (PDGFRs) or receptor tyrosine kinases (RTKs) are transmembrane proteins consisting of five extracellular immunoglobulin-like domains and a cleaved intracellular tyrosine kinase domain. Two isoforms of the PDGFR chain form dimers upon binding of the PDGF ligand, which leads to three possible combinations of dimers: αα, -ββ, and -αβ [109].

PDGFR can also bind VEGF. Recent studies have shown that all PDGF dimers, with the exception of PDGF-DD, bind to receptor 2 of the vascular endothelial growth factor VEGFR2 [110]. The structural similarities between PDGF and VEGF and their ligand-receptor interactions indicate their strong functional relationship. PDGFR activation leads to intracellular signaling pathways, including Ras/Rac, MAPK, PI3K, STA Src, and others, which subsequently promote proliferation and migration [104].

SNVs have been found in the *PDGF* and *PDGFR* genes, but not all of them have associations with the disease.

Zhou et al. investigated the association of the *PDGF* gene SNVs with or near susceptibility to vascular disease in a Native American population with a high prevalence of systemic sclerosis. Both genes encoding PDGF receptors (*PDGFRA* and *PDGFRB)* showed no associations with cardiovascular diseases [111].

Joosten et al. investigated the associations between polymorphic sites in the human *PDGFRA* gene promoter and neural tube defects. Five different haplotypes have been identified. Two of these (H1/H2) were overrepresented in patients with sporadic spina bifida and were associated with increased PDGFRA transcriptional activity. Together with PDGFR-α knockout or knockout results, these results indicate that the correct level of PDGFR-α signaling is critical for proper neural arches development, suggesting that both too high and too low levels of PDGFR-α expression or signaling can lead to spina bifida [112].

## 4. Discussion

**Figure 6:**
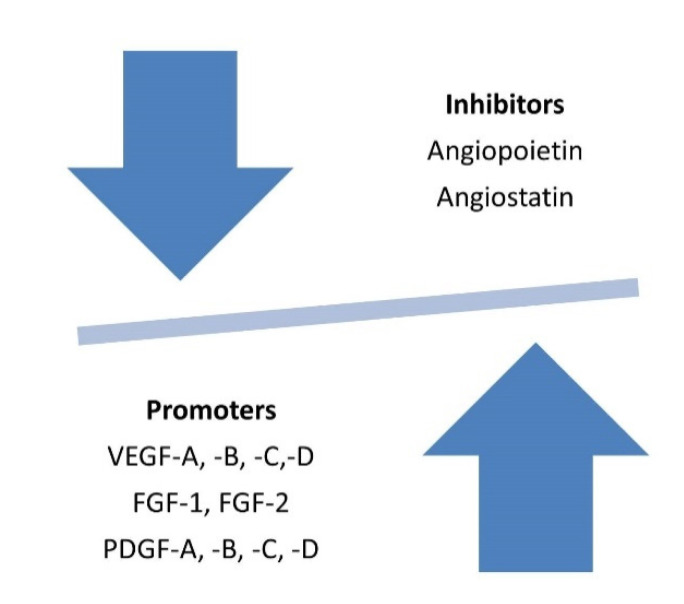
A balance of promoters (proangiogenic factors) and inhibitors (anti-angiogenic factors) of angiogenesis in an athlete’s body (brain, myocardium, lung, and skeletal muscle).

Angiogenesis is a complex process of formation of new blood capillaries (Figure 6). The creation of a new vascular network is realized through the activation of endothelial cells, the transformation of proteinases in them, degradation of the extracellular structure of the tissue, proliferation, and movement of cellular structures. Physiological angiogenesis, as a rule, is the body’s response to hormonal changes (angiogenesis in the reproductive system) or to environmental factors, primarily hypoxia.

Recent studies show that the main stimulus in the processes of balancing the synthesis of proangiogenic and anti-angiogenic molecules or “angiogenic switch” is played by an oxygen-sensitive protein complex with transcriptional activity—hypoxia-inducible factor 1-alpha (HIF- 1α) [7]. It is considered the leading transcriptional regulator of mammalian genes responsible for the response to a lack of oxygen.

HIF-1α activation occurs at physiologically important sites of oxygen pathway regulation, providing fast and adequate responses to hypoxic stress, including the expression of genes that regulate the process of angiogenesis [8,9,12,16]. At the same time, it is necessary to understand that sport training is a powerful epigenetic factor that can cause multidirectional changes in an athlete’s body, especially in early teens and adolescents. An important aspect of adaptation to training loads in cyclic sports is an increase in the number of capillaries in muscle fibers, which improves the metabolism of skeletal muscles and myocardium as well as in nervous and lung tissue.

Physical activity is accompanied by functional, electrical, and structural remodeling of the heart. Myocardial hypertrophy is accompanied by an increase in the diameter of the left atrium, wall thickness of the left ventricle, and myocardial mass. Vascular changes in the myocardium are associated with changes in the density of capillaries in the heart muscle [13]. Angiogenesis in the myocardium is a complex process. Cardiomyocytes express receptors for growth factors, which are rapidly activated in response to hypoxia [14]. It is believed that the role of growth factors in myocardial remodeling is related to the balance of its actions on receptors that inhibit the development of hypertrophy and receptors that have prohypertrophic effects. Thus, among the factors contributing to the transition from adaptive to pathological myocardial remodeling, a significant role belongs to the “angiogenic switch” imbalance [ibid.]. In recent years, a significant amount of knowledge has been accumulated on the regulation of the formation of new vessels in the hypertrophied heart. It became clear that myocardial endothelial cells not only respond to hemodynamic forces and paracrine signals from neighboring cells, but also actively participate in the processes of cardiac remodeling, stimulating the growth and contractility of cardiomyocytes or the production of extracellular matrix proteins in myofibroblasts. Moreover, in response to adequate signals, they can change their phenotype and transdifferentiate into extracellular cells that produce matrix. As myocardial vascularization plays a central role in the transition from adaptive heart hypertrophy to heart failure, further study of the signaling mechanisms involved in the regulation of angiogenesis in the myocardium is important in sports practice [ibid.].

Multiple isoforms of growth factors generated by alternative splicing can play different roles in vascular development and stimulate various cellular functions, including migration, proliferation, and differentiation [75]. The study of the “angiogenic switch” problem in the cerebrovascular and cardiovascular systems showed that the formation of new vessels is mediated by a complex interaction of all growth factors. In addition, a complex hierarchical control is indicated through the transmission of VEGF signals to other growth factors [82].

Sports loads in cyclic sports are associated with an increase in oxygen consumption, so the pulmonary system becomes the main system subject to functional changes during exercise. Numerous studies of the physiology of sports have demonstrated a clear relationship between heart rate, oxygen consumption, respiratory rate and minute ventilation, energy metabolism, and lactate production [35]. Although the lungs are one of the limiting systems of the body in cyclic sports, their response to high-intensity loads and other environmental stresses is often overlooked. Airway epithelial cells are the predominant source of several growth factors throughout lung organogenesis and appear to be critical for normal alveolarization, rapid alveolar proliferation, and normal vascular development. Pulmonary growth factors are synthesized by alveolar epithelial cells, bronchial epithelial cells, smooth muscle cells, and alveolar macrophages [41]. The lung is one of the main organs in which growth factors control several important physiological functions [37]. There are many controversial questions about the role of growth factors in the physiology and pathology of the lungs. The presented review has demonstrated that when playing sports, it is necessary to carefully consider the possible positive and negative effects of growth factors on the lung tissue. The most studied is the role of VEGF-A in the physiological development of lung tissue. The influence of other growth factors remains to be seen [38].

Primarily, the “angiogenic switch” is important in aerobic sports (such as long distance running). The high capillarization of the working muscles increases the peripheral circulation and thus influences the maximum oxygen consumption. Increasing maximum oxygen consumption is a key objective in enhancing athletic performance in long distance runners (endurance training). This determines the relevance of studying angiogenic mechanisms in skeletal muscles, myocardium, lung, and nervous tissue during training.

It seems promising to study the activity of expression of genes encoding the process of angiogenesis in representatives of cyclic sports. It was found that the severity of the angiogenic process depends on the intensity of physical activity, which indicates the adaptive nature of the process of angiogenesis. This confirms the point of view, according to which the change in the “angiogenic switch” stroke is a marker of athletes’ physical performance.

The importance of different isoforms of growth factors, their signaling pathways involved in angiogenesis of skeletal muscles, myocardium, cerebrovascular and cardiovascular systems, and lung tissue induced by exercise remains to be elucidated [90]. Vascular endothelial growth factor-B (VEGF-B) and angiopoietin 1 (ANGPT-1) are the main angiogenic factors that enhance endothelial cell proliferation and accelerate vascular growth in the infarction zone [103]. There is information on the effect of VEGF-A on endothelial cells as a mitogen through repeated endothelialization. It is believed that VEGF-B, VEGF-C, VEGF-D, and PlGF also play a role in physiological angiogenesis, but have not been extensively studied. Four isoforms of FGF (FGF1, FGF2, FGF4, and FGF6) are the most studied as proangiogenic factors. PDGF-A and PDGF-B polypeptide chains are well studied as regulators of several physiological and pathophysiological processes, mainly using cells of mesenchymal or neuroectodermal origin as targets. Over the past decade, two additional ligands have been identified for two PDGF receptors (PDGF-C and PDGF-D). Their role in the angiogenic process is still poorly understood. It has been suggested that cell signaling mediated by growth factors is more complex than previously thought.

## 5. Conclusions

Angiogenesis is the physiological process of the formation of new blood capillaries, which play an important role in the functioning of skeletal muscles, myocardium as well as lung and nervous tissue in athletes. Violation of the “angiogenic switch” as a balance between proangiogenic and anti-angiogenic molecules can lead to a decrease in the functional resources of the nervous, musculoskeletal, cardiovascular, and respiratory systems in athletes and, as a consequence, to a decrease in sports performance.
